# Rhinovirus Infection Drives Complex Host Airway Molecular Responses in Children With Cystic Fibrosis

**DOI:** 10.3389/fimmu.2020.01327

**Published:** 2020-07-16

**Authors:** Kak-Ming Ling, Luke W. Garratt, Erin E. Gill, Amy H. Y. Lee, Patricia Agudelo-Romero, Erika N. Sutanto, Thomas Iosifidis, Tim Rosenow, Stuart E. Turvey, Timo Lassmann, Robert E. W. Hancock, Anthony Kicic, Stephen M. Stick

**Affiliations:** ^1^Paediatrics, Medical School, Faculty of Healthy and Medical Science, The University of Western Australia, Nedlands, WA, Australia; ^2^Telethon Kids Institute, Respiratory Research Centre, Nedlands, WA, Australia; ^3^Telethon Kids Institute, Centre for Health Research, The University of Western Australia, Nedlands, WA, Australia; ^4^School of Biomedical Sciences, The University of Western Australia, Nedlands, WA, Australia; ^5^Centre for Microbial Diseases and Immunity Research, University of British Columbia, Vancouver, BC, Canada; ^6^Department of Pediatrics, BC Children's Hospital, University of British Columbia, Vancouver, BC, Canada; ^7^Occupation and Environment, School of Public Health, Curtin University, Perth, WA, Australia; ^8^Department of Respiratory and Sleep Medicine, Perth Children's Hospital, Nedlands, WA, Australia; ^9^Centre for Cell Therapy and Regenerative Medicine, School of Medicine and Pharmacology, The University of Western Australia, Nedlands, WA, Australia; ^10^St. John of God Hospital, Subiaco, WA, Australia; ^11^Priority Research Centre for Asthma and Respiratory Disease, Hunter Medical Research Institute, Newcastle, NSW, Australia; ^12^Robinson Research Institute, University of Adelaide, North Adelaide, SA, Australia; ^13^Murdoch Children's Research Institute, Melbourne, VIC, Australia; ^14^Department of Paediatrics, University of Melbourne, Melbourne, VIC, Australia

**Keywords:** cystic fibrosis, RV, airway epithelial cells, transcriptomic, innate immune response

## Abstract

Early-life viral infections are responsible for pulmonary exacerbations that can contribute to disease progression in young children with cystic fibrosis (CF). The most common respiratory viruses detected in the CF airway are human rhinoviruses (RV), and augmented airway inflammation in CF has been attributed to dysregulated airway epithelial responses although evidence has been conflicting. Here, we exposed airway epithelial cells from children with and without CF to RV *in vitro*. Using RNA-Seq, we profiled the transcriptomic differences of CF and non-CF airway epithelial cells at baseline and in response to RV. There were only modest differences between CF and non-CF cells at baseline. In response to RV, there were 1,442 and 896 differentially expressed genes in CF and non-CF airway epithelial cells, respectively. The core antiviral responses in CF and non-CF airway epithelial cells were mediated through interferon signaling although type 1 and 3 interferon signaling, when measured, were reduced in CF airway epithelial cells following viral challenge consistent with previous reports. The transcriptional responses in CF airway epithelial cells were more complex than in non-CF airway epithelial cells with diverse over-represented biological pathways, such as cytokine signaling and metabolic and biosynthetic pathways. Network analysis highlighted that the differentially expressed genes of CF airway epithelial cells' transcriptional responses were highly interconnected and formed a more complex network than observed in non-CF airway epithelial cells. We corroborate observations in fully differentiated air–liquid interface (ALI) cultures, identifying genes involved in IL-1 signaling and mucin glycosylation that are only dysregulated in the CF airway epithelial response to RV infection. These data provide novel insights into the CF airway epithelial cells' responses to RV infection and highlight potential pathways that could be targeted to improve antiviral and anti-inflammatory responses in CF.

## Introduction

Lung disease is the major cause of morbidity and mortality in cystic fibrosis (CF) (1). Progressive lung damage is associated with mucus obstruction, neutrophilic inflammation, and chronic airway infection and is already evident in the first years of life (2–6). Intermittent pulmonary exacerbations occur in individuals with CF who experience increased respiratory symptoms and reduction in pulmonary function that are responsive to therapy with antibiotics (7). Moreover, the frequency of exacerbations is a predictor of long-term morbidity and irreversible loss of lung function (8, 9). The triggers for these pulmonary exacerbations are not fully understood although it is recognized that lower respiratory infections caused by viruses are likely to play a significant role (10–14).

The most common virus detected in the airway of adults and children with CF is human rhinovirus (RV) (15–19). The clinical impact of RV includes reduction of lung function/FEV_1_ (15, 20, 21), hospitalization (22), and increased requirement for intravenous antibiotic treatment (11, 14). Recent longitudinal data suggest that RV infection persists for a longer period in individuals with CF compared to non-CF controls (14), a finding consistent with *in vitro* observations that suggest a defective innate response of epithelial cells to RV (23, 24). The nature of any intrinsic deficiency still remains unclear although some explanations are now emerging (25).

In this study, we hypothesized that the antiviral responses of primary airway epithelial cells (AEC) from children with CF are dysregulated following RV infection. We utilized transcriptome sequencing (RNA-Seq) to assess the gene expression of CF (ΔPhe508del homozygous) and non-CF primary AEC pre- and post-RV infection. Differential expression analysis was carried out to compare the antiviral responses between CF and non-CF AEC. Functional analyses identified diverse biological pathways and complex networks in response to RV infection in CF AEC that were less apparent in non-CF AEC. We performed additional work to validate some of these unique biological pathways using primary differentiated AEC culture models, and data corroborates observations made from the RNA-Seq analysis. Overall, this study provides insights into the global transcriptomic response by non-CF and CF AEC to RV infection and has identified potential therapeutic targets that could reduce the harmful contribution of RV to progressive lung disease in individuals with CF.

## Materials and Methods

### Patient Recruitment and Establishment of Primary Bronchial Epithelial Cells

The study was approved by the St. John of Gods Human Ethics Committee (SJOG#901) and Perth Children's Hospital Ethics Committee (#1762), and written informed consent was obtained from parents or guardians. Children without CF were recruited prior to undergoing elective surgery for non-respiratory-related conditions. Children with CF and homozygous for the Phe508del mutation were recruited during annual early surveillance visits (2, 3, 23). Subject demographic data for RNA-Seq analysis are provided in Table 1. Samples were obtained by brushing of the tracheal mucosa of children using a cytology brush as previously described (23, 26). Submerged monolayer primary airway epithelial (AEC) cultures from non-CF children and those with CF were then established, expanded in Bronchial Epithelial Basal Medium (BEBM® LONZA™), supplemented with growth additives and 2% (v/v) Ultroser G (Pall Corporation) (23, 26–28), and used for experimentation. Subject demographic data for the validation experiments are provided in Table 2. Here, primary AECs were differentiated into ciliated pseudostratified AECs as described previously (29). Briefly, AECs were initially seeded on 0.4-μm polyester membrane culture inserts grown to confluence (Corning, NY, USA) and ALI cultures established. These were maintained for 28 days, and both beating cilia and mucus production were well-established. Prior to ALI validation experiments, inserts were confirmed to have a transepithelial electrical resistance (TEER) measurement >800 Ω/cm^2^.

**Table 1:** Patient demographic for subjects used for RNA sequencing analysis including five non-CF children and seven children with CF.

	**Non-CF control^#^**	**Cystic fibrosis**
Number of participants	5	7
Mean Age ± sd (yr)	3.5 ± 1.4	2.8 ± 2.3
Age range (yrs)	(1.7–5.4)	(0.2–5.6)
Male (%)	40	57
Genotype	Healthy non-CF	p. Phe508del/ p. Phe508del
NE Activity (%)	NA	43
IL-8 Detected in BALs (%)	NA	100
Microorganisms detected in BALf (%)	NA	14 (*Pseudomonas aeruginosa*)
PRAGMA Disease (%)	NA	3.44(2.24–4.16)

# *Non-CF control were children who underwent elective surgery for non-respiratory-related conditions*.

**Table 2:** Patient demographics for subjects used for validation work including six non-CF children and six children with CF.

	**Non-CF control^#^**	**Cystic fibrosis**
Number of participants	6	6
Mean Age ± sd (yr)	3.3 ± 0.65	2.3 ± 2.3
Age range (yrs)	(2.4-4.0)	(0.2–5.9)
Male (%)	50	83
Genotype	Healthy non-CF	p. Phe508del/ p. Phe508del
NE Activity (%)	NA	50
IL-8 Detected in BALs (%)	NA	100
Microorganisms detected in BALf (%)	NA	50
PRAGMA Disease (%)	NA	3.53

# * Non-CF control were children who underwent elective surgery for non-respiratory related conditions*.

### Human RV Infection and RNA Extraction

To emulate an acute RV infection episode *in vitro*, we exposed AEC with RV1b (courtesy of P. Wark, University of Newcastle) at MOI 12.5 (23, 30, 31). After 24 h, culture supernatant was collected for cytokine measurement and cell pellets for RNA extraction. RNA was extracted using a PureLink® RNA (Life Technologies) mini kit as per manufacturer instructions. Total RNA was eluted with 30 μL RNase free water with the addition of 1 μL of RNase Inhibitor (Life Technologies). RNA purity and yield were determined using a NanoDrop, and integrity was assessed using an Agilent RNA 6000 Nanochip on an Agilent Bioanalyser.

### RNA Sequencing (RNA-Seq) and Analysis

Samples identified with high purity (1.8–2.0 range A260/280) and quality (RIN > 8.0) were then processed for library preparation. Here, the KAPA Stranded mRNA-Seq kit (KAPABiosystems) was used for mRNA capture and fragmentation (~200–300 bp fragments). RNA fragments were then subsequently reverse transcribed into cDNA strands, followed by adapter ligation and library amplification. Sequencing of these libraries (100 bp, single-end) was performed on the Illumina HiSeq 2500 platform at an average depth of 5.08 ± 1.17 million reads (Figure S1A) per. The quality and quantity of the FASTQ sequence reads were assessed using FastQC (v0.11.3) (32), followed by mapping to the reference genome (Homo sapiens hg19/GRCh37 – Ensembl) using “hisat” (v0.1.6-beta) (33). Gene-level quantification (counts) of hisat alignments was performed using SummarizeOverlaps and, finally, post-alignment QC using Samstat (v1.5.2.) (34). Mapping rates to the human genome were within the expected rate for all samples at 88.2–91.2% (35), and post-alignment quality control using SAMStat 1.5.2 reported an average high quality (mapping quality score of thirty) mapping rate of 89.98% ± 0.67 (Figure S1B).

### Bioinformatics and Statistical Analysis

Bioinformatics and statistical analyses were performed on five non-CF and seven CF samples. Statistical analysis was conducted in PRISM 8 (v8.1.2; GraphPad Software Inc., California, USA) and included the Mann–Whitney test to compare the statistical variance between genotype, and the Wilcoxon test was used to compare the statistical difference between paired samples. All subsequent bioinformatic analyses post-alignment were performed in R (v3.4.1) (36). To remove low-abundance genes, only those that had a minimum of 10 counts per sample in at least five or more samples were included, resulting in a total of 12,757 genes analyzed. The R package RUVseq (1.10.0) (37) was applied to normalize RNA-Seq read counts between samples to remove the unwanted variance. Differential gene expression was determined using DESeq2(v1.16.1) (38) after calculating variance-stabilizing transformation (VST) from the fitted dispersion mean relation to yield count data with constant variance along the range of mean values. We determined those genes with an adjusted *p*-value ≤ 0.05 and ± 1.5-fold change as statistically and biologically significant, respectively. To visualize the variance between samples, a principal component analysis plot was generated using the plotPCA function in DESeq2 and visualized using ggplot2 (v3.1.0) (39). Next, we identified non-infected baseline non-CF and CF enriched gene ontology (GO) terms from the biological process (BP) using Metascape (http://metascape.org) (40). Visualization of GO term analysis was performed using the GOPlot (v1.0.2) (41). The GoCircle function was used to highlight gene expression changes within each of the selected terms. The value of the *z*-score from GOPlot is calculated as zscore = (up - down) ÷ √count, where up and down were the number of up- and down-regulated genes respectively.

### Pathway Analysis and Protein–Protein Interaction Network-Based Enrichment Analysis

Pathway analysis based upon Reactome repositories was performed using Signature Over-Representation Analysis (SIGORA) version 2.0.1. The pathway enrichment by SIGORA was identified according to statistically over-represented Pathway Gene-Pair Signatures (Pathway-GPS) (42). To expose the interactive associations among the DEGs at the protein level, genes obtained from both non-CF and CF responses were mapped using protein–protein interactions (PPI) via NetworkAnalyst (http://www.networkanalyst.ca/). Network Analyst (43, 44) and was based upon IMEX Interactome, a comprehensive, high-quality protein–protein interaction database curated from InnateDB (45) to characterize the relationships and interactions of input genes. The network was built by limiting the original seed proteins only and picking zero order interactions.

### ELISA

Cytokine production of interleukin 8 (IL-8) (Becton Dickinson, Biosciences, San Diego, CA), interferon lambda 1, 2, 3 (IFNλ1, λ2, λ3), RANTES (CCL5), interleukin (IL)-1B, Interferon gamma-induced protein 10 (IP-10) (R&D, MN, Minneapolis) in culture supernatant was measured by ELISA. Production of interleukin 6 IL-6 was measured using a time-resolved fluorometry detection system (PerkinElmer, Waltham, MA). Expression of interferon beta (IFNβ) was measured using an AlphaLISA® bead-based assay (PerkinElmer).

### Corroboration of RNA-Seq Observations in Fully Differentiated Cultures

Experiments were then performed to assess whether unique pathways identified from the initial RNA-Seq analysis were evident in fully differentiated 3-D cultures. Primary ALI cultures were established and, upon TEER confirmation, were rinsed three times with sterile room temperature 1× phosphate-buffered saline for 10 min at 37°C. Cultures were then subsequently infected with RV1b at MOI 0.1 in 200 μL for 24 h. An MOI of 0.1 was chosen based on the lowest infection dose from the literature with no reported cytopathic effects or major disruption to the epithelium (46–48) as this prevents assessment of mucin-related enzymes and other downstream analyses. After 24 h, inserts were harvested in RNA lysis buffer for RNA extraction using the PureLink® RNA (Life Technologies) mini kit as per manufacturer instructions. Total RNA was eluted with 30 μL RNase free water. Genes were chosen from at least two independent pathways identified to be uniquely expressed by AEC in children with CF in response to RV infection and included *IL1R2, STS8SIA4, ST6GALNAC2, MAN1A1*, and *B3GNT8*. Gene expression was determined via real-time qRT-PCR (refer to Supplementary Materials and Methods 1.1 and 1.2) using TaqMan® pre-designed primer/probes (ThermoFisher Scientific). Details on all primer probes are listed in Table S8.

## Results

### Patient Demographics

**Figure 1:**
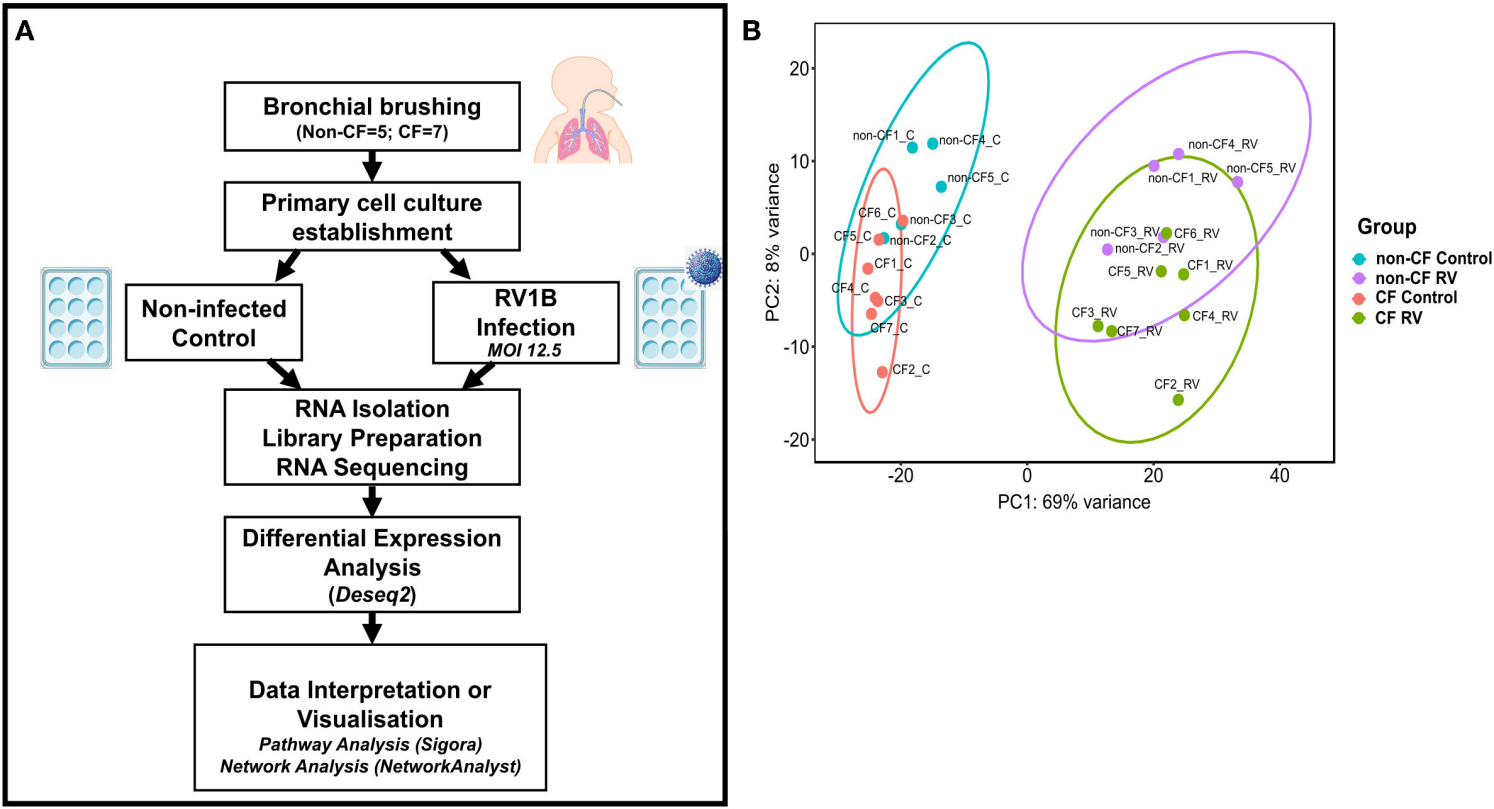
Analytical methods. **(A)** Schematic workflow describing experimental procedure and transcriptomic analysis. Primary airway epithelial cells (AECs) from non-CF children (*n* = 5) and children with CF (*n* = 7) were obtained from bronchial brushings. AECs were established for infection with human rhinovirus 1B (RV1B) at MOI 12.5. At 24 h post-infection, RNA was isolated and processed for library preparation. Sample libraries were sequenced on the Illumina HiSeq 2500 platform as described in the methods section **(B)** Principal component analysis (PCA). Components 1 (PC1) and 2 (PC2) highlight distinct clustering of samples. PC1 shows the highest percentage of variance (69%) for all samples and completely separates the control and RV-infected samples. PC2 shows the second highest variance (8%) and separates non-CF and CF samples. Data points represent individual samples for non-CF controls (turquoise), non-CF infected (purple), CF control (coral), and CF infected (green).

The demographic information of children participating in this study is shown in Table 1 (RNA sequencing) and Table S1 (entire sample sets, including those additional samples used for ELISA). Non-CF controls were children who underwent elective surgery for non-respiratory-related conditions and did not possess existing lung disease. RNA samples of primary AECs obtained from these children (*n* = 32, 16 non-CF and 16 CF) were originally collected, both pre- and post-infection with RV *in vitro*. RNA sequencing was performed on all samples as summarized in the workflow diagram (Figure 1A). A sample elimination process was carried out to exclude unqualified samples (detailed in Figure S3). Samples that were run on a different sequencer did not pass rigorous quality control for RNA sequencing (mapping quality score >30, *n* = 3), and those with sequencing depth of less than one million reads (*n* = 9) were excluded from analysis. Finally, RNA sequencing samples from a total of seven CF and five non-CF children were included for the differential expression analysis by applying a fold change cutoff of ≥1.5-fold. Only one child with CF had detectable microorganisms in bronchioalveolar lavage fluid (BALf) during the time of AEC sampling. The PRAGMA CT score presented as percentage of disease was also conducted to demonstrate the quantitative measurement of disease progression during the time of sampling in children with CF.

### Distinct Transcriptional Changes of AEC in Response to RV Infection

The normalized read counts matrix was used to build a non-supervised principal component analysis to visualize the major contributors to transcriptional variation within this data set (Figure 1B). The first principal component (PC1, 69% of the variance) completely separated RV-infected and non-infected AEC, and separation of uninfected or infected CF and non-CF AEC was observed on PC2 (8% of the variance), indicating that patient genotype is the second largest source of variation within the data set.

### Modest Transcriptional Differences Between Uninfected CF and Non-CF AEC

**Figure 2:**
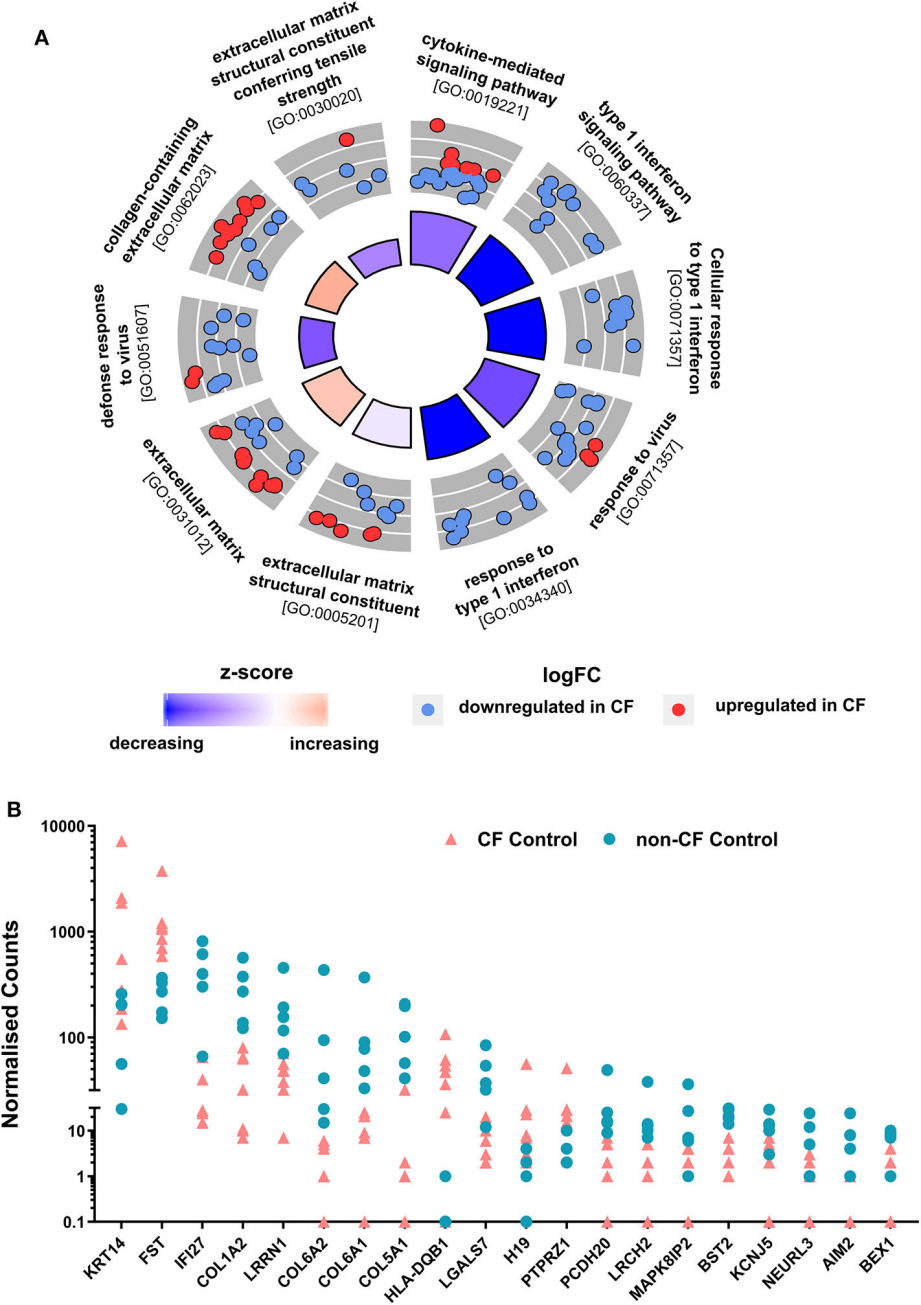
Comparison of non-CF and CF non-infected baseline control. **(A)** Circular visualization of gene-annotation enrichment analysis of non-infected baseline samples. Statistically significant differentially expressed genes (DEGs) between non-infected CF and non-CF samples were annotated using gene ontology (GO). The circular plot combines gene expression and gene-annotation enrichment data. The outer circle shows a scatterplot for each enriched GO term of the Log2FC of the assigned genes. Red dots indicate upregulation, and blue dots indicate downregulation in CF non-infected control compared to non-CF. The inner ring is a bar plot where the height of the bar indicates the significance of GO terms (log10-adjusted *p*-value), and color corresponds to the *z*-score: blue, decreased; red, increased; and white, unchanged. **(B)** Normalized gene counts of the top 20 DEGs between CF and non-CF non-infected baseline samples with the highest fold change, data points represent individual samples for non-CF controls (turquoise; circle) and CF control (coral; triangle).

To determine whether the AECs transcriptional profiles from children with CF are intrinsically differed from non-CF controls, non-infected baseline CF and non-CF AECs were analyzed for differential gene expression. We observed a total of 162 DEGs with absolute fold change ≥1.5 between non-infected baseline CF and non-CF AECs. Among those, 92 genes were significantly downregulated, and 70 genes were significantly upregulated in CF AEC compared to non-CF AEC. To identify in which biological processes the 162 DEGs were involved, we performed gene ontology (GO) term enrichment analysis (41). The predominant enriched GO term in CF AEC is depicted by a circle plot (Figure 2A). The circle plot highlights the overall gene expression change by showing increased expression in red and decreased expression in blue. The *p*-value of the GO terms is represented by the height of the inner rectangle, which is also colored by *z*-score based on GOPlot formula (zscore = ([number of up-regulated genes] − [number of down-regulated genes]) ÷√[gene count]). Analysis identified the cytokine-mediated signaling pathway and type 1 interferon signaling pathway as the top enriched GO terms with decreased *z*-score and extracellular matrix as the GO term with an increased *z*-score. The full list of the top upregulated and downregulated genes is summarized in Table S2. The top DEG from differential expression analysis comparing non-infected baseline CF and non-CF AEC was *HLA-DQB1* (HLA Class II GWAS genes). We also identified the top 20 genes with the highest fold change between non-infected CF and non-CF AEC (Figure 2B). These genes were found to be involved in biological processes including type 1 interferon signaling pathway (*AIM2, BST2, IFI27*), keratin (*KRT14*), DNA methylation (*H19*), cell cycle (*BEX1*), extracellular matrix (*COL1A2, COL5A1, COL6A1, COL6A2*), cell–cell interaction (*LGALS7*), signal transduction (*FST, LRCH2, LRRN1*), calcium ion binding (*PCDH20*), potassium channel (*KCNJ5*), transferase activity (*NEURL3*), and phosphatase activity (*PTPRZ1*).

### CF AEC Have More Transcriptional Changes in Response to RV Infection Than Non-CF AEC

**Figure 3:**
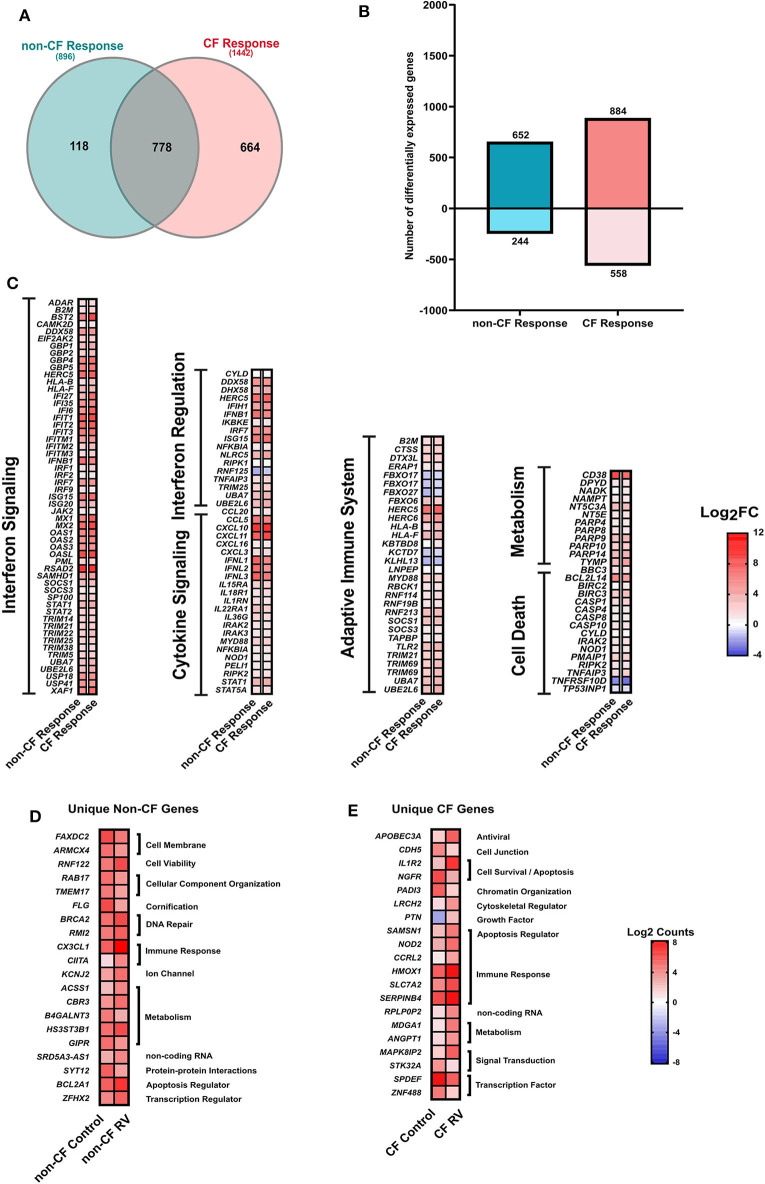
Response to rhinovirus infection. **(A)** Venn diagram comparing the differentially expressed (DEGs) genes between non-CF (teal; *non-CF RV-infected* vs. *non-CF non-infected control*) and CF (pink; *CF RV-infected* vs. *CF non-infected control*) response to RV1B infection. A total of 778 DEGs were common between non-CF and CF response to RV1B infection. **(B)** The number (*y*-axis) and direction of change (upregulated = positive *y*-axis, downregulated = negative *y*-axis) of DEGs (|Log_2_FC|1.5, adjusted *p*-value < 0.05) of non-CF and CF response to rhinovirus infection (*x*-axis). **(C)** The relative expression genes that are commonly differentially expressed (|Log_2_FC|>1.5, adjusted *p*-value < 0.05) in airway epithelial cells (AECs) from non-CF and CF individuals when infected with rhinovirus. These genes are associated with immune response, including interferon signaling, cytokine signaling, adaptive immune system, cell death, and metabolism. **(D)** The normalized counts (Log_2_Counts) of the top 20 genes that are uniquely differentially in AECs from non-CF children when infected with rhinovirus, including genes associated with cellular component, DNA repsir, immune response, ion channel and activity, cellular metabolism, protein–protein interactions and regulation of apoptotic process. **(E)** The normalized counts (Log_2_Counts) of the top 20 genes that are uniquely differentially expressed in AECs from children with CF when infected with rhinovirus, these genes are involved in the apoptotic process, cell–cell junction, chromatin organization, cytoskeletal regulator, growth factor, immune response, cellular metabolism, signal transduction, and transcription regulation.

We next analyzed the RNA-Seq data to assess the transcriptomic response of CF and non-CF AECs collected after infection with RV. Comparative analysis of response profiles indicates that AECs from both CF and non-CF differentially modulated the expression of several genes related to the innate antiviral immune response in response to RV infection. The Venn diagram (Figure 3A) was used to compare genes that were uniquely and commonly modulated between CF response (RV-infected CF AEC vs. uninfected CF AEC) and non-CF response (RV-infected non-CF AEC vs. uninfected non-CF AEC) to RV infection. A total of 896 (652 upregulated, 244 downregulated) DEGs were observed in the non-CF response to RV and 1442 DEGs (884 upregulated, 558 downregulated) in the CF response (Figures 3A,B). Candidate genes were ranked according to their extent of differential expression when compared to uninfected samples. Although there was considerable overlap between the groups (778 common DEGs, Figure 3C), there were significantly more unique DEGs (Figures 3D,E) specific to the CF response (664) compared with the non-CF response (118). A majority of overlapping DEGs were involved in the core immune response to RV infection, including interferon signaling, interferon regulation, cytokine signaling, cell death, and metabolism. The unique DEGs for both CF and non-CF AEC in response to RV infection are summarized (Tables S3, S4, respectively). The top unique DEG for the non-CF response was *CX3CL1*, which is an important chemoattractant to attract other immune cells, such as dendritic cells. Other top unique genes for the non-CF response were found to be associated with the cellular component *(FAXDC2, ARMCX4, RAB17, TMEM17*), DNA repair (*BRCA2, RMI2*), and cellular metabolism (*CBR3, B4GALNT3, HS3ST3B1, GIPR*). Nevertheless, 46% (664 out of 1442) of DEGs for the CF AEC response to RV infection were found to be unique with the *IL-1R2* gene, the IL-1 signaling decoy receptor, being the top unique DEG (4.8-fold change). Other unique genes for the CF AEC response were found to be associated with growth factor (*PTN*), immune response (*NOD2, CCRL2, HMOX1, SLC7A2, SERPINB4*), cellular metabolism *(MDGA1, ANGPT1*), cytoskeletal regulation (*LRCH2*), signal transduction (*MAPK8IP2, STK32A*), and transcription regulation (*SPDEF, ZNF488*).

### RV Infection Drives Common Epithelium-Induced Innate Antiviral Response in CF and Non-CF AEC

Genes that were commonly modulated in CF and non-CF AECs (Table S5) were found to be key drivers of core epithelium-induced innate antiviral response to RV infection. Specifically, RV infection triggered a significant upregulation of type I and III interferons (*IFNB1, IFNL1, IFNL2, IFNL3*) in both CF and non-CF AECs (Figure 3C). However, it was evident that the fold changes (Log_2_FC) of *IFNB1* (5.8-fold), *IFNL1* (5.8-fold), *IFNL2* (5.1-fold), and *IFNL3* (6.1-fold) in gene expression in response to RV infection were lower in the CF AEC response compared to the non-CF AEC response (*IFNB1*: 6.9-fold, *IFNL1*: 7.2-fold, *IFNL2*: 7-fold, *IFNL3*: 7.5-fold). Interferon signaling also triggered the induction of a variety of interferon-stimulated genes (ISGs), including *Mx1*; viperin (*RSDA2*); and the IFITM, IFIT, and OAS family in both CF and non-CF AECs (Figure 3C).

**Figure 4:**
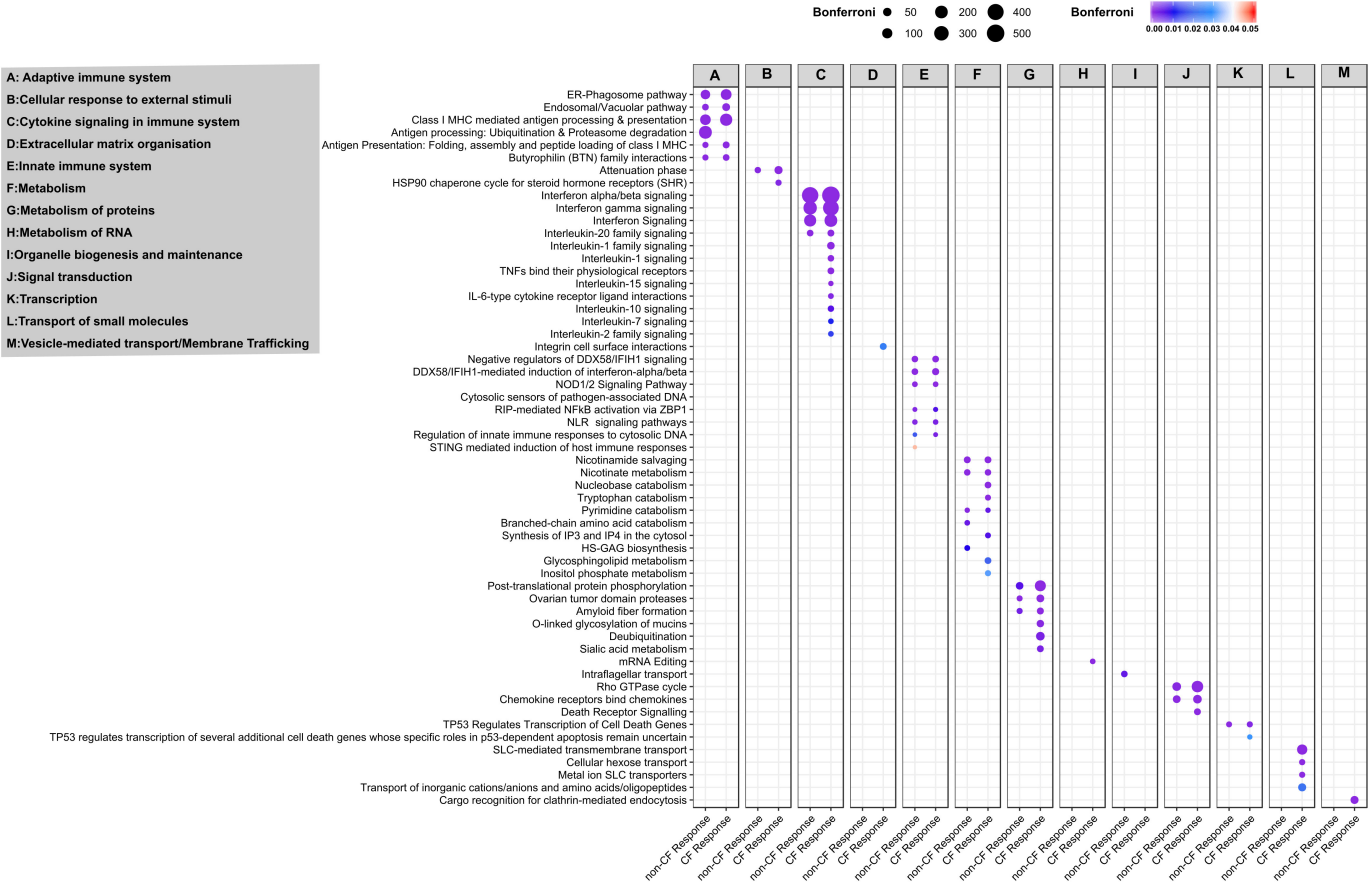
Pathway and network analysis of non-CF and CF AECs response to rhinovirus infection. Pathway enrichment analysis results from 896 and 1,442 differentially expressed genes in non-CF and CF response to rhinovirus infection, respectively. Figure depicting the association of common and unique enriched pathways using Reactome database with the differentially expressed gene lists based on SIGORA successes metric (circle size) and the color bar depicting the significance of the association (Bonferroni < 0.05). The enriched biological pathways are categorized according to functions **(A)**, adaptive immuno system, **(B)** cellular response to external stimuli, **(C)** cytokine signaling in immune system, **(D)** extracellular matric organization, **(E)** innate immune system, **(F)** metabolism, **(G)** metabolism of proteins, **(H)** metabolism of RNA, **(I)** organelle biogenesis and maintenance, **(J)** signal transduction, **(K)** transcription, **(L)** transport of small molecules and **(M)** vesicle-mediated transport/membrane trafficking.

We extended our analysis to identify the biological pathways corresponding to all DEGs in CF and non-CF AECs in response to RV infection. The full list of enriched biological pathways for CF and non-CF AECs' antiviral responses are provided in Tables S6, S7, respectively. SIGORA pathway analysis was then performed using gene-pair signature pathway analysis, which only accounts for statistically significant gene pairs unique to the over-represented pathways. This analysis identified 52 and 31 biological pathways responsible for CF and non-CF AEC host responses to RV infection, respectively. Comparing the two, we identified 26 common significantly enriched biological pathways (Figure 4), which are mainly categorized into five main functions, including (1) cytokine signaling in the immune system, (2) presentation to the adaptive immune system, (3) innate immune system, (4) metabolism or biosynthetic, and (5) signal transduction. Consistently, the core antiviral response was demonstrated by type I and III interferon and other antiviral factors as reported earlier with interferon-α/β signaling, interferon-γ signaling, and interferon signaling being the top three most enriched pathways associated with cytokine signaling. Other common cytokine responses, such as interleukin 20 family signaling (Figure 4A, Table S6), was over-represented with upregulation of *IL22RA1*, STAT family (*STAT1, 2, 3*, and *5A*), JAK family (*JAK1, JAK2*), and the negative regulator of IFN signaling *SOCS3*. Infection with RV has also significantly increased gene expression of chemokines such as *CXCL10, CXCL11, CXCL3, CXCL16, CCL2, CCL5*, and *CCL20* in both CF and non-CF AECs. Additionally, we also detected transcriptional changes in pathogen recognition receptors, such as *TLR3, DDX58 (RIG-I)* and *IFIH (MDA5)*, and other key genes that regulate innate immune signaling, including *IKBKE, IRF7, ISG15, NFKBIA, UBE2L6, UBA7*, and *DDX58*. Genes involved in the over-represented pathway Class I MHC-mediated antigen processing and presentation, such as the gene set of F-box protein, TRIM, and the HERC family were the common DEGs in response to RV infection. The PARP protein family, including *PARP4, PARP8, PARP9, PARP10*, and *PARP14*, responsible for the regulation of nicotinamide metabolism and salvaging of cellular redox reactions, were also upregulated in response to RV infection. The changes of genes involved in nucleotide biosynthesis pathway pyrimidine catabolism were observed, including *NT5C3A, DPYD, TYMP*, and *NT5E*. Changes in gene expression of caspases (*CASP1, 4, 8, 10*), which provide pivotal links in cell regulatory networks controlling inflammation and cell death, were also observed in both CF and non-CF AEC post-RV infection.

### CF AEC Transcriptome Reveals More Biological Pathways and a More Complex Network in Response to RV Infection Than for Non-CF AEC

In addition to the common over-represented pathways induced by RV infection, we observed an additional 26 enriched pathways specific to the CF response (Figure 4A). In addition to the five functions mentioned above, the unique over-represented pathways also fall under another two functions, including extracellular matrix organization and vesicle-mediated transport or transport of small molecules. Additional pathways categorized in cytokine signaling in the immune system, such as interleukin 1, 2, 7, 10, and 15 signaling pathways, were the unique enriched pathways specific to CF response. Genes associated with interleukin 1 family signaling–driven proinflammatory activity are *IL36G*; receptor antagonist *IL36RN*; *IL1R2*; *IL1RN*; receptor *IL18R1*; protein phosphatase *PTPN12*; pellino proteins *PELI1, PELI3*, and IRAK kinase *IRAK2, IRAK3*; and key immune and inflammatory response regulator *S100A12*. Other cytokines with essential immunomodulatory functions, including IL-7, IL-10, IL-15, and IL-2 family signaling, were the significantly over-represented pathways unique for CF response to RV infection. Furthermore, we observed a significant upregulation of the chemotactic factors for neutrophils *CXCL1* and *CXCL2* in the CF AEC response to RV infection. Downregulation of genes encoding E3 ubiquitin ligases, such as *TRIM45* (regulator of TNFα-induced NF-κB-mediated transcriptional activity) and *RNF128* (inhibitor of cytokine gene transcription), were also only observed in the CF response. The transcriptional change of the *HSPA5* gene was also observed in the CF response as part of major histocompatibility complex (MHC) class I molecules mediated adaptive immune regulation.

Several metabolism/biosynthetic pathways of notable interest to CF airway disease include nucleobase catabolism, inositol phosphate metabolism, synthesis of IP3 and IP4 in the cytosol and tryptophan catabolism, which were all altered in CF response to RV infection (Figure 4A). We observed transcriptional changes of ectonucleotidases in the nucleobase catabolism pathways, particularly ecto-nucleoside triphosphate diphosphohydrolases (ENTPDases) *ENTPD3* (downregulated) and *ENTPD6* (upregulated). The inositol phosphate metabolism pathway was also found to be altered in CF AECs, namely the downregulation of genes encoding phosphohydrolases *NUDT11*, phospholipase *PLCH2* and *PLCD4*, kinase *ITPKB*, and phosphatase *INPP4B*. We also observed a group of upregulated genes, including *KYNU, KMO, IDO1, AADAT*, and *CCBL1*, which are associated with the key biosynthetic process of tryptophan catabolism. Biological pathways regulating metabolism of proteins, notably mucin metabolism (O-linked Glycosylation of mucins and sialic acid metabolism), were also over-represented pathways for the CF response. Additionally, RV infection in CF AEC triggered transcriptional changes of transport of small molecules (including cellular hexose transport, metal ion SLC transporters, transport of amino acids, and SLC-mediated transmembrane transport). We noted transcriptional changes for genes involved in extracellular matrix organization, such as integrin α5 and β6 (*ITGA5, ITGA6*) and cell adhesion molecule *ICAM1*.

To better understand the potential functional interaction of DEGs, we also visualized expression and investigated the underlying molecular interactions between genes by generating zero-order PPI subnetworks (Figures S2A,B). The main CF and non-CF PPI subnetwork consisted of functionally enriched pathways that play imperative roles in the host antiviral response to RV infection. The non-CF AEC response subnetwork identified associations of 254 nodes and 565 edges (Figure S2A). We observed 172 genes with a degree more than one interactor, where 27 nodes were observed with ≥10 connections with other nodes. Key hub genes regulating the antiviral response found included *STAT1, STAT2, IRF2, IRF1, ISG15, DDX58, IRF7, RIPK1, IKBKE*, and *CASP8*. Conversely, a more complex CF AEC response subnetwork projected the associations of 493 nodes and 1156 edges (Figure S2B). We observed 320 genes with a degree more than one, where 66 nodes were observed with ≥10 connections with other nodes. The key hub genes regulating the CF AEC response subnetwork included *IRF1, ISG15, STAT1, STAT3, HSAP1B, CASP8, TBK1, IKBKE, TRAF2*, and *CASP8*. The key hub genes of both CF and non-CF subnetworks are represented by key regulators related to the innate immune system and cytokine signaling.

### Aberrant Cytokine Production of CF AECs to RV Infection

**Figure 5:**
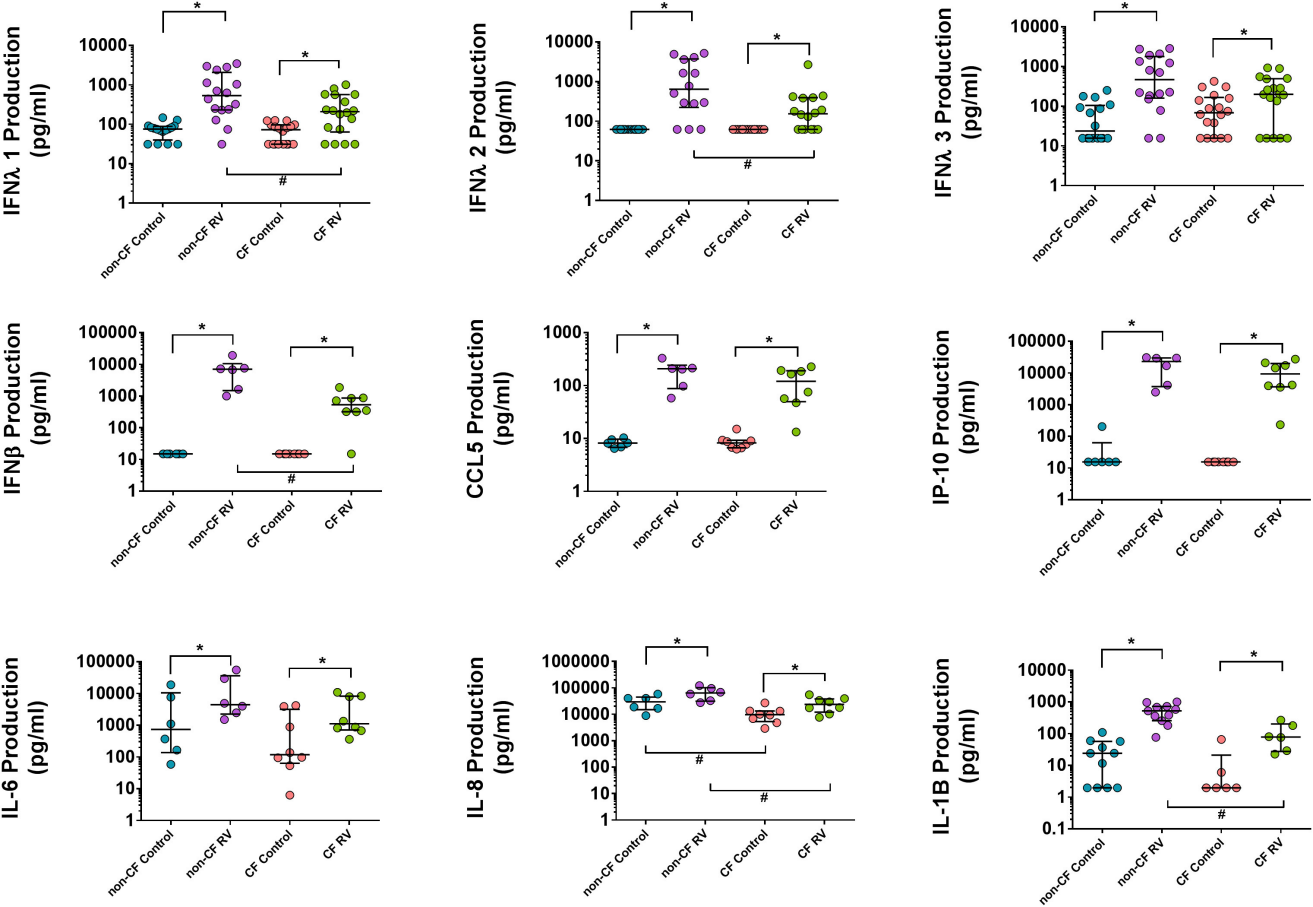
Cytokine production in the supernatant AEC of non-CF individuals and children with CF following RV infection. Cytokine release was measured in cell culture supernatants using commercial ELISA kits and an in-house time-resolved fluorometry detection system. Type 1 and III interferons (IFN-β, IFN-λ1, and IFN-λ2) were significantly higher in non-CF AEC post-RV infection compared to CF AEC. Inflammatory cytokines IL-6, IL-8, and IL-1B were significantly increased in both CF and non-CF RV-infected samples with significantly higher IL-8 and IL-1B levels produced by non-CF RV-infected samples compared to CF RV-infected samples. RANTES (CCL5) and IP-10 were significantly elevated in CF and non-CF RV-infected samples but not significant between phenotype. Note: *n* = 9–11 for non-CF and 6–12 for CF. The data were represented as median ± IQR, symbols show statistical significance in RV-infected samples relative to paired non-infected control samples; *p < 0.05, determined using Wilcoxon test. Statistically significant for comparison between CF and non-CF non-infected samples determined using unpaired *t*-test or Mann–Whitney depending on Gaussian distribution, ^#^*p* < 0.05.

In order to validate the transcriptional changes of the enriched cytokine signaling pathways, we measured the levels of key innate and inflammatory cytokine production at 24 h post-RV infection (Figure 5). Although *IFNB1* was significantly induced upon RV infection in both cohorts, this is not reflected at the protein level with significantly lower levels (average 10.8-fold) of IFNβ1 (type 1 interferon) released by CF AEC (668.3 ± 576.2 pg/ml; *p* < 0.05) compared to non-CF AEC (7,265 ± 6,558 pg/ml). As shown in Figure 3C, all type 3 IFN genes (*IFNL1, IFNL2, IFNL2*) were upregulated post RV infection. Cytokine levels of the type III interferons IFNλ1, IFNλ2, and IFNλ3 were also significantly elevated in both CF and non-CF AEC infected with rhinovirus. However, levels of IFNλ1 (296.4 ± 293.3 pg/ml) and IFNλ2 (334.6 ± 642.8 pg/ml) produced by CF AEC in response to RV infection were significantly (3.5- to 5-fold) lower when compared to non-CF AEC (1,059 ± 1,170 pg/ml and 1,665 ± 1,932 pg/ml, respectively; *p* < 0.05). IFNλ3 produced by CF AEC (285.3 ± 287.3 pg/ml) following RV infection was somewhat but nonsignificantly lower compared to that produced by non-CF subject AEC (928.6 ± 997.9 pg/ml). Similar cytokine levels of antiviral chemokines CCL5 (RANTES) and IP10 and pro-inflammatory cytokines, including IL6 were detected in non-infected CF and non-CF AECs, and similar increases in these proteins occurred in response to RV infection. However, IL-8 and IL-1β cytokine production is significantly elevated in non-CF AECs in response to RV infection compared to CF AECs.

### Corroboration of Unique Gene Expression Patterns in Response to RV Infection in CF ALI Cultures

**Figure 6:**
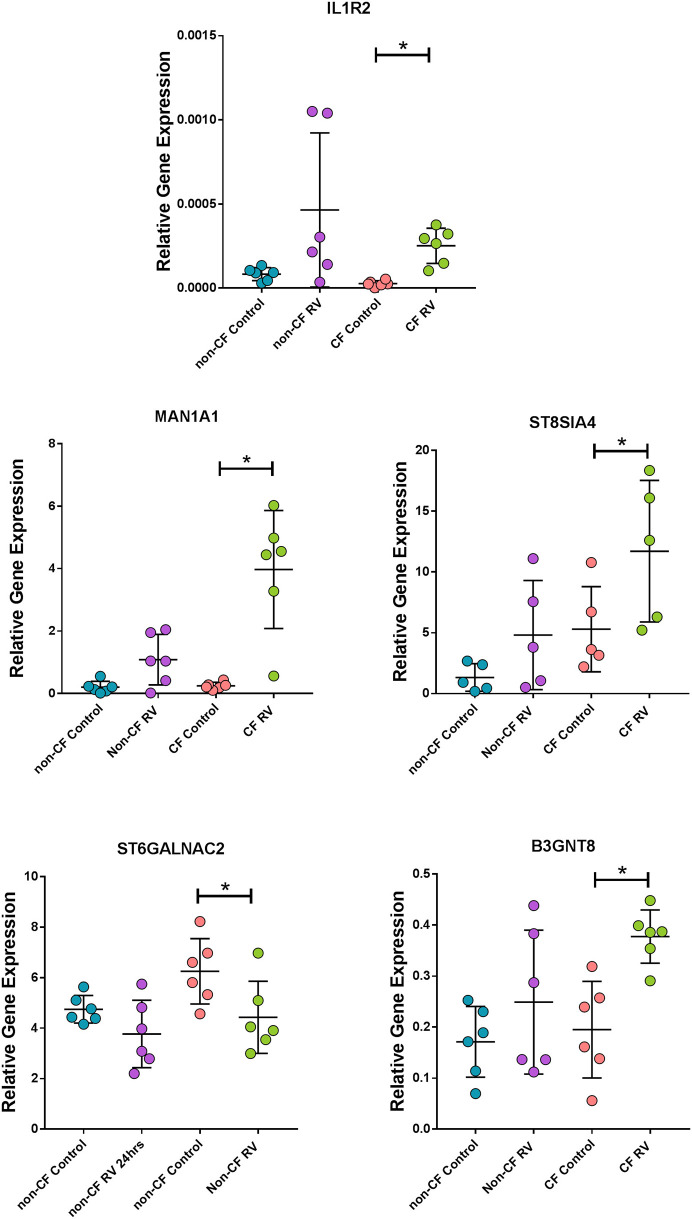
Corroboration of uniquely expressed genes by CF AEC in fully differentiated cultures. Gene expression of the top unique gene *IL1R2* as well as mannosidase (*MAN1A1*), sialyltransferases (*ST8SIA4* and *ST6GALNAC2*), and acetylglucosaminyltransferase (*B3GNT8*), were uniquely altered in CF air–liquid interface cultures when infected with rhinovirus (RV) for 24 h. Data points represent individual samples for non-CF controls (turquoise), non-CF infected (purple), CF control (coral), and CF infected (green). Expression of all genes were normalized to the housekeeping gene PPIA (49). *Indicates *p* < 0.05 by one-way ANOVA following normal distribution test.

To validate results generated from the RNA-Seq in a model that better represents the airway, we assessed the expression of some unique DEGs identified in submerged CF cultures post- RV infection by challenging ALI cultures with the same RV and again assessing gene expression at 24 h (Figure 6). Expression of the top unique DEG for the CF response, *IL1R2*, was validated with a consistent increase in CF ALI post RV infection (9.4-fold over uninfected, *p* < 0.05; Figure 6). Upregulation of *IL1R2* appeared bimodal in non-CF ALI and was not significant (*p* = 0.30). Furthermore, expression of genes involved in glycosylation of mucins and sialic acid metabolisms, namely sialyltransferase *ST8SIA4, ST6GALNAC2*, mannosidase *MAN1A1*, and acetylglucosaminyltransferase *B3GNT8*, was also validated as unique to CF (Figure 6). A significantly higher level of sialyltransferase *ST8SIA4* expression (2.2-fold, *p* < 0.05) was observed in RV-infected CF ALI cultures while *ST6GALNAC2* was significantly downregulated (−1.4-fold, *p* < 0.05). The mannosidase *MAN1A1* were dramatically upregulated by 16.3-fold (*p* < 0.05) in CF ALI at 24 h post RV infection. *B3GNT8*, an acetylglucosaminyltransferase that adds N-acetylglucosamine (GlcNAc) to N-glycans was also increased by 1.9-fold (*p* < 0.05) in CF ALI in response to RV infection. Expressional changes of these genes were all consistent with the RNA-Seq data from submerged cultures. We observed that these genes did not change expression in non-CF ALI cultures upon infection with RV; *ST8SIA4* (3.6-fold, *p* = 0.14); *ST6GALNAC2* (−1.3-fold, *p* = 0.09); *MAN1A1* (5.4-fold; *p* = 0.08); *B3GNT8* (1-fold, *p* = 0.28).

## Discussion

To improve knowledge of the underlying epithelial transcriptional responses during infection with rhinovirus, a major respiratory pathogen, we performed RNA sequencing on primary AEC from children with CF and non-CF controls *in vitro* at baseline and post-RV infection. There are five important findings from this study: (i) There were only modest baseline transcriptional differences between non-infected CF and non-CF AECs prior to exposure to RV, (ii) there was conservation in certain core antiviral responses (e.g., IFN signaling) of CF and non-CF AECs at the transcriptomic level but not the protein level, (iii) CF AECs elicited a larger and more complex transcriptional response compared to non-CF AECs with multiple unique biological pathways represented, (iv) key among these biological pathways are cytokine signaling and biosynthetic pathways (e.g., O-linked glycosylation of mucins) as they are highly relevant to CF lung pathology, (v) we corroborated observations made from the RNA-Seq analysis in fully differentiated cultures and identified genes involved in IL-1 signaling and mucin glycosylation that are only dysregulated in the CF airway epithelial response to RV infection. Collectively, these results identify potential biological pathways and processes that could be contributing to the adverse outcomes typically seen in people with CF during virus infection.

There were only modest baseline transcriptional differences between non-CF and CF AECs. This is most likely reflective of the very early and mild lung disease in the CF cohort. Minimal baseline differences also provide confidence that any difference in the antiviral transcriptional changes that we observed were due to infection. Nevertheless, the top differentially expressed baseline was HLA-DQB1, previously identified in a GWAS study with a high association signal to CF lung disease severity [reviewed in (50)]. Interestingly, the major enriched GO terms for the differentially expressed genes in non-infected baseline samples were denoted by the cytokine-mediated signaling pathway and type 1 interferon signaling pathway. Among these, *AIM2* inflammasome (associated with induction of pyroptosis, activation of pro-inflammatory cytokines, and viral suppression) (51, 52) *IFI27* (also known as *ISG12a*) contributes to IFN-dependent perturbation of normal mitochondrial function and enhanced cellular apoptosis (53), and the IFN-dependent antiviral factor *BST2* were all significantly downregulated in CF AECs. In response to RV infection, several common responses were found, including interferon signaling. However, the induction of type 1 and 3 interferon genes was lower in CF AEC. This was mirrored by reduced type 1 (IFNβ1) and type 3 interferon (IFNλ1 and IFNλ2) protein in supernatant. The reduction of type 1 and 3 interferon production of CF AECs in response to RV infection could be associated with the negative regulation of interferon signaling by the unique key gene, such as STAT3 (54, 55); however, this requires further characterization. Conversely, the IL-1 family signaling pathway was unique to the CF AECs response to RV infection, but in this case, IL-1β protein was significantly lower in CF supernatant compared to non-CF. This unusual observation could be, in part, mediated by negative regulators of IL-1 signaling expressed in CF AEC, including *IL1R2* and *IL1RN*, pellino protein genes *PELI1* and *PELI3*, together with interleukin 1 receptor-associated kinase *IRAK2* and *IRAK3*. We then assessed the expression of *IL1R2* pre- and post-RV infection in a differentiated culture model and made similar observations to those obtained using submerged cultures.

The IL-1 signaling pathway has been suggested as a link between hypoxic cell death and sterile neutrophilic inflammation in CF (56). Both IL-1α and IL-1β were detectable in bronchioalveolar lavage fluids (BALs) of young children with CF in the absence of bacterial infection, highlighting potential for inflammation of the CF airway under sterile inflammation (57). Since *S100A12* (key regulator of inflammatory process) is also part of the IL-1 family signaling pathway in CF response to RV infection, we postulate that the CF AECs could be directing from pro-inflammatory IL-1 signaling under sterile inflammation to a hyperinflammatory condition characterized by NF-κB signaling cascades during RV infection. Other evidence suggests the alteration of the inflammatory response with abundant cytokine signaling pathways (interleukin 1, 2, 7, 10, and 15 signaling) in CF AEC post-RV infection could be explained by downregulation of *RNF128* genes, which functions as an inhibitor of cytokine gene transcription and could interact with TBK1 (key hub of CF AEC response in our study here) kinase activity to enhance antiviral immunity. We also observed an elevated IL-8 production in both CF and non-CF AECs post-RV infection with higher amounts produced by non-CF AECs compared to CF AECs. Our IL-8 results agree with a previous study that also utilized primary AEC cultures in a similar infection setting (58) but contrasts with another that observed elevated inflammatory mediator release by the CF AECs (23). Overall, the over-represented cytokine signaling pathways suggest a unique and prominent role in regulating inflammation in CF AECs when infected with RV. However, with conflicting observations in this area, elucidating the complexity of the inflammatory response with associated cell death in CF AECs warrants further investigation.

We also identified over-represented metabolic pathways in CF AEC in response to RV infection specifically involved in the regulation of immunity, including inositol phosphate metabolism and synthesis of IP3 and IP4 in the cytosol, suggesting an altered CF airway microenvironment after RV infection. The induction of inositol phosphate has previously been related to endoplasmic reticulum expansion and Ca^2+^ storage, resulting in Ca^2+^-dependent transcriptional activity of inflammatory mediators (59), which could contribute to hyperinflammatory responses seen in the CF AEC to viral infection (23). Upregulation of extracellular ectonucleotidase in the inositol phosphate metabolism pathway was found to cause depletion of ATP concentrations, reduction of air–surface liquid volume, ASL collapse, and failure in mucociliary clearance may trigger CF lung disease exacerbations as shown previously in a model of respiratory syncytial virus infection (60). Another metabolic pathway, trytophan catabolism, was also one of the over-represented pathways in the CF AECs following RV infection. Tryptophan metabolism has been previously found to be dysregulated in CF AEC (61) and has been implicated in *Pseudomonas aeruginosa* infection, oxidative stress, and Th17 hyperinflammation (62, 63). Alteration of tryptophan metabolism results in accumulation of kynurenine and anthranilate, which could subsequently disrupt the homeostatic balance of the host's innate immune system and reduce the antimicrobial activity of airway epithelium.

Other identified biosynthetic pathways associated with RV infection in CF include sialic acid metabolism and O-linked glycosylation of mucins. Sialic acids are a family of negatively charged monosaccharides that are commonly expressed as the terminal residues in glycans of the glycoconjugates on the epithelial cell surface lining the airways and are also major components of secreted mucins in the airway. Previous studies have identified increased fucoslyation and decreased sialylation in cultured AEC while a contrary observation was reported in CF sputum (64–67). As a key player that contributes to the rheological properties of mucus, aberrant sialic acid metabolism may worsen the pathological conditions of CF. O-linked glycosylation is a post-translational modification process and occurs within the endoplasmic reticulum (ER) and Golgi complex. The enzymes in ER and Golgi complex regulate glycosylation of N-glycans and O-glycans by successively adding to and then remodeling mucin oligosaccharides prior to transport to cell membranes for tethering or secretion. Here, alteration of genes encoding glycosyltransferases, such as N-acetylgalactosaminyltransferases, N-acetlyglucosaminyltransferase, and galactosyltransferases, were reported from our RNA-Seq analysis. We corroborated a number of these as unique to the AEC response to RV in children with CF. Changes in these glycosyltransferases could potentially alter the O-glycans on cell surfaces and, thus, affect interactions with airway pathogens and irritant exposures. Emerging evidence suggests alteration of mucin glycosylation is a response to infection and inflammation and might induce extended conformational changes to prevent damage from proteolytic enzymes (68). Although the impact that *CFTR* mutations has on mucin biomolecules is unknown, our results suggest that RV infection could be a potential mechanism that contributes to changes in mucin glycosylation that are exclusive to CF and that might influence mucosal barrier function. A previous investigation has demonstrated that a surplus of unfolded proteins that results from blocked glycosylation leads to prolonged ER stress and activation of the unfolded protein response (UPR) causing cell death (69). Previous *in vitro* work using an immortalized cell line discovered a pronounced reprogramming of host cell metabolism toward an anabolic state, including upregulation of glucose uptake, glycogenolysis, nucleotide synthesis, and lipogenesis (70). Considering most of the metabolic changes found in this study occur post-RV infection, future studies integrating the transcriptomic signature patterns with analyses of the metabolites produced by CF AEC in response to RV infection will provide significant insight into the exact metabolic changes that occur during infection.

Interestingly, RV infection in CF AECs results in the upregulation of a group of SLC transporter genes, including upregulation of *CP* (ferroxidase), *SLC41A2* (magnesium transporter), *SLC30A1* (zinc transporter), and *SLC39A8* (zinc transporter) and downregulation of *SLC39A10* (zinc transporter) and *SLC40A1* (iron-regulated transporter). Increasing total iron and zinc has previously been associated with airway inflammation in CF (71). These results suggest that RV infection in the CF airway is associated with the presence of redox active biometals. A previous study (72) has suggested that the dysregulation of iron homeostasis is accompanied by a respiratory virus infection, which, in turn, facilitates pseudomonas biofilm growth. Understanding the mechanistic link of virus infection to the alteration of the cellular microenvironment and instigation of secondary infection might aid in development of new treatment.

We acknowledge some limitations in the experimental design. First, we only analyzed transcriptomics at the 24-h time point, primarily due to the limited number and expansion of primary cells established from each patient. However, early optimization of our infection model did assess the transcriptional changes earlier (data not shown), and the greatest transcriptional change identified occurred at the 24-h time point. Although methodologies now exist to assist with primary AEC expansion *in vitro* (29), its effects at the transcriptomic level remain unknown and, thus, the use of unaltered primary airway cells remains a significant strength of this study. Future investigations could possibly include additional time points to better appreciate the transcriptional signature changes over the full course of RV infection as well as the long-term consequence of viral infection on CF AECs. Second, this study utilized a laboratory strain of rhinovirus (RV1b), which might exert differential effects on CF AEC compared to clinically derived isolates known to cause exacerbations in this cohort. With different RV serotypes causing infection in CF airways (10), future studies may identify whether innate immune responses may be serotype-specific. Similarly, comparison studies to other viruses (respiratory syncytial virus, influenza) would also assist in our understanding of the contribution of early-life viruses to CF disease progression. Finally, the simplified monolayer cell culture model of basal CF AECs may be regarded as a limitation, but basal cells are the primary target of RV (73). While monolayer cultures may oversimplify the multicellular interactions of epithelial (ciliated, goblet, basal, secretory cells) and immune cells (dendritic cells, neutrophils), it is an important, repeatable model with low methodological variation, and we were able to validate genes in differentiated AEC. Overall, we are highly confident that limitations are minor and that our results provide new insight into new therapeutic targets for treating acute viral infections in CF that can be validated in future transcriptomic studies assessing differentiated AEC models.

In conclusion, this study shows that, at the transcriptomic level, CF AECs induce a complex and unique set of responses when infected with RV *in vitro* that have implications for lung disease progression in CF. Despite type 1, II, and III interferon signaling being involved in the core CF antiviral response, IFNs protein levels were lower in CF AEC when compared to non-CF AEC. Metabolic and biosynthetic pathways were unexpectedly integrated with the core CF antiviral response, and multiple key regulatory molecules of antiviral response were dysregulated in CF AEC, revealing new potential to modulate CF AEC innate immunity to RV infection. Future work will explore whether these regulatory molecules are potential targets for therapy unique to RV and may be leveraged to reduce the impact viral infections have on lung disease progression CF.

## Data Availability Statement

Raw datasets have been uploaded to GEO, with accession number GSE138167.

## Ethics Statement

The study was approved by the St. John of Gods Human Ethics Committee (SJOG#901) and Perth Children's Hospital Ethics Committee (#1762) and written informed consent was obtained from parents or guardians.

## Author Contributions

Conceptualization: SS and AK. Funding acquisition: SS, AK, RH, and ST. Methodology: K-ML, EG, AL, TL, PA-R, TI, and TR. Supervision: SS, AK, TL, and LG. Validation: K-ML, ES, and LG. Manuscript writing: K-ML, LG, and AK. Manuscript review: SS, RH, TL, PA-R, ES, ST, TI, and EG. All authors contributed to the article and approved the submitted version.

## Conflict of Interest

The authors declare that the research was conducted in the absence of any commercial or financial relationships that could be construed as a potential conflict of interest.

## References

[1] Cutting GR. Cystic fibrosis genetics: from molecular understanding to clinical application. Nat Rev Genet. (2015) 16:45–56. 10.1038/nrg3849 25404111 PMC4364438

[2] Sly PD Brennan S Gangell C De Klerk N Murray C Mott L. Lung disease at diagnosis in infants with cystic fibrosis detected by newborn screening. Am J Respir Crit Care Med. (2009) 180:146–52. 10.1164/rccm.200901-0069OC 19372250

[3] Stick SM Brennan S Murray C Douglas T von Ungern-Sternberg BS Garratt LW. Bronchiectasis in infants and preschool children diagnosed with cystic fibrosis after newborn screening. J Pediatr. (2009) 155:623–8.e1. 10.1016/j.jpeds.2009.05.005 19616787

[4] Sly PD Gangell CL Chen L Ware RS Ranganathan S Mott LS. Risk factors for bronchiectasis in children with cystic fibrosis. N Engl J Med. (2013) 368:1963–70. 10.1056/NEJMoa1301725 23692169

[5] Tang AC Turvey SE Alves MP Regamey N Tümmler B Hartl D. Current concepts: host-pathogen interactions in cystic fibrosis airways disease. Eur Respir Rev. (2014) 23:320–32. 10.1183/09059180.00006113 25176968 PMC9487317

[6] Esther CR Muhlebach MS Ehre C Hill DB Wolfgang MC Kesimer M. Mucus accumulation in the lungs precedes structural changes and infection in children with cystic fibrosis. Sci Transl Med. (2019) 11:eaav3488. 10.1126/scitranslmed.aav3488 30944166 PMC6566903

[7] Fennell PB Quante J Wilson K Boyle M Strunk R Ferkol T. Use of high-dose ibuprofen in a pediatric cystic fibrosis center. J Cyst Fibros. (2007) 6:153–8. 10.1016/j.jcf.2006.06.003 16844429

[8] Sanders DB Bittner RCL Rosenfeld M Hoffman LR Redding GJ Goss CH. Failure to recover to baseline pulmonary function after cystic fibrosis pulmonary exacerbation. Am J Respir Crit Care Med. (2010) 182:627–32. 10.1164/rccm.200909-1421OC 20463179 PMC5450763

[9] Asner S Waters V Solomon M Yau Y Richardson SE Grasemann H. Role of respiratory viruses in pulmonary exacerbations in children with cystic fibrosis. J Cyst Fibros. (2012) 11:433–9. 10.1016/j.jcf.2012.04.006 22579414 PMC7105203

[10] de Almeida MB Zerbinati RM Tateno AF Oliveira CM Romão RM Rodrigues JC. Rhinovirus C and respiratory exacerbations in children with cystic fibrosis. Emerg Infect Dis. (2010) 16:996–9. 10.3201/eid1606.100063 20507756 PMC3086221

[11] Kieninger E Singer F Tapparel C Alves MP Latzin P Tan HL. High rhinovirus burden in lower airways of children with cystic fibrosis. Chest. (2013) 143:782–90. 10.1378/chest.12-0954 23188200

[12] Goffard A Lambert V Salleron J Herwegh S Engelmann I Pinel C. Virus and cystic fibrosis: rhinoviruses are associated with exacerbations in adult patients. J Clin Virol. (2014) 60:147–53. 10.1016/j.jcv.2014.02.005 24637203 PMC7108260

[13] Stelzer-Braid S Johal H Skilbeck K Steller A Alsubie H Tovey E. Detection of viral and bacterial respiratory pathogens in patients with cystic fibrosis. J Virol Methods. (2012) 186:109–12. 10.1016/j.jviromet.2012.08.008 22940004

[14] Dijkema JS Ewijk BE van Wilbrink B Wolfs TFW Kimpen JLL Ent CK van der. Frequency and duration of rhinovirus infections in children with cystic fibrosis and healthy controls: a longitudinal cohort study. Pediatr Infect Dis J. (2016) 35:379–83. 10.1097/INF.0000000000001014 26658528

[15] Flight WG Bright-Thomas RJ Tilston P Mutton KJ Guiver M Morris J. Incidence and clinical impact of respiratory viruses in adults with cystic fibrosis. Thorax. (2014) 69:247–53. 10.1136/thoraxjnl-2013-204000 24127019

[16] Shah A Connelly M Whitaker P McIntyre C Etherington C Denton M. Pathogenicity of individual rhinovirus species during exacerbations of cystic fibrosis. Eur Respir J. (2015) 45:1748–51. 10.1183/09031936.00229114 25976682

[17] Esposito S Daleno C Scala A Castellazzi L Terranova L Sferrazza Papa S. Impact of rhinovirus nasopharyngeal viral load and viremia on severity of respiratory infections in children. Eur J Clin Microbiol Infect Dis. (2014) 33:41–48. 10.1007/s10096-013-1926-5 23893065 PMC7088146

[18] Gangell CL Shackleton C Poreddy S Kappers J Gaydon JE Sloots TP. Feasibility of parental collected nasal swabs for virus detection in young children with cystic fibrosis. J Cyst Fibros. (2014) 13:661–6. 10.1016/j.jcf.2014.02.009 24637444 PMC7105194

[19] Deschamp AR Hatch JE Slaven JE Gebregziabher N Storch G Hall GL. Early respiratory viral infections in infants with cystic fibrosis. J Cyst Fibros. (2019) 18:844–50. 10.1016/j.jcf.2019.02.004 30826285 PMC6711838

[20] Etherington C Naseer R Conway SP Whitaker P Denton M Peckham DG. The role of respiratory viruses in adult patients with cystic fibrosis receiving intravenous antibiotics for a pulmonary exacerbation. J Cyst Fibros. (2014) 13:49–55. 10.1016/j.jcf.2013.06.004 23891398

[21] Cousin M Molinari N Foulongne V Caimmi D Vachier I Abely M. Rhinovirus-associated pulmonary exacerbations show a lack of FEV 1 improvement in children with cystic fibrosis. Influenza Other Respi Viruses. (2016) 10:109–12. 10.1111/irv.12353 26493783 PMC4746558

[22] Armstrong D Grimwood K Carlin JB Carzino R Hull J Olinsky A. Severe viral respiratory infections in infants with cystic fibrosis. Pediatr Pulmonol. (1998) 26:371–9. 10.1002/(sici)1099-0496(199812)26:6<371::aid-ppul1>3.0.co;2-n 9888211

[23] Sutanto EN Kicic A Foo CJ Stevens PT Mullane D Knight DA. Innate inflammatory responses of pediatric cystic fibrosis airway epithelial cells: effects of nonviral and viral stimulation. Am J Respir Cell Mol Biol. (2011) 44:761–7. 10.1165/rcmb.2010-0368OC 21317379

[24] Schögler A Stokes AB Casaulta C Regamey N Edwards MR Johnston SL. Interferon response of the cystic fibrosis bronchial epithelium to major and minor group rhinovirus infection. J Cyst Fibros. (2016) 15:332–9. 10.1016/j.jcf.2015.10.013 26613982 PMC7185532

[25] Schögler A Caliaro O Brügger M Oliveira Esteves BI Nita I Gazdhar A. Modulation of the unfolded protein response pathway as an antiviral approach in airway epithelial cells. Antiviral Res. (2019) 162:44–50. 10.1016/j.antiviral.2018.12.007 30550797

[26] Kicic A Sutanto EN Stevens PT Knight DA Stick SM. Intrinsic biochemical and functional differences in bronchial epithelial cells of children with asthma. Am J Respir Crit Care Med. (2006) 174:1110–18. 10.1164/rccm.200603-392OC 16908868

[27] Lane C Burgess S Kicic A Knight DA Stick S. The use of non-bronchoscopic brushings to study the paediatric airway. Respir Res. (2005) 6:53. 10.1186/1465-9921-6-53 15943866 PMC1180854

[28] McNamara PS Kicic A Sutanto EN Stevens PT Stick SM. Comparison of techniques for obtaining lower airway epithelial cells from children. Eur Respir J. (2008) 32:763–8. 10.1183/09031936.00162507 18757700

[29] Martinovich KM Iosifidis T Buckley AG Looi K Ling KM Sutanto EN. Conditionally reprogrammed primary airway epithelial cells maintain morphology, lineage and disease specific functional characteristics. Sci Rep. (2017) 7:17971. 10.1038/s41598-017-17952-4 29269735 PMC5740081

[30] Garratt LW Sutanto EN Foo CJ Ling KM Looi K Kicic-Starcevich E. Determinants of culture success in an airway epithelium sampling program of young children with cystic fibrosis. Exp Lung Res. (2014) 40:447–59. 10.3109/01902148.2014.946631 25191759

[31] Kicic A Stevens PT Sutanto EN Kicic-Starcevich E Ling K-M Looi K. Impaired airway epithelial cell responses from children with asthma to rhinoviral infection. Clin Exp Allergy. (2016) 46:1441–55. 10.1111/cea.12767 27238549

[32] Andrews S FastQC A Quality Control tool for High Throughput Sequence Data. (2017) Available online at: https://www.bioinformatics.babraham.ac.uk/projects/fastqc/ (accessed June 01, 2017).

[33] Kim D Langmead B Salzberg S. HISAT: hierarchical indexing for spliced alignment of transcripts. Nat Methods. (2014) 12:357–60. 10.1101/01259125751142

[34] Lassmann T Hayashizaki Y Daub CO. SAMStat: monitoring biases in next generation sequencing data. Bioinformatics. (2011) 27:130–31. 10.1093/bioinformatics/btq614 21088025 PMC3008642

[35] Conesa A Madrigal P Tarazona S Gomez-Cabrero D Cervera A McPherson A A survey of best practices for RNA-seq data analysis. Genome Biol. (2016) 17:13 10.1186/s13059-016-0881-8 26813401 PMC4728800

[36] Dalgaard P R Development Core Team: R: A Language and Environment for Statistical Computing (2010).

[37] Risso D Ngai J Speed TP Dudoit S Normalization of RNA-seq data using factor analysis of control genes or samples. Nat Biotechnol. (2014) 32:896–902. 10.1038/nbt.2931 25150836 PMC4404308

[38] Love MI Huber W Anders S. Moderated estimation of fold change and dispersion for RNA-seq data with DESeq2. Genome Biol. (2014) 15:550. 10.1186/s13059-014-0550-8 25516281 PMC4302049

[39] Wickham H Ggplot2 : Elegant Graphics for Data Analysis. New York, NY: Springer-Verlag (2016). Available online at: https://ggplot2.tidyverse.org (accessed June 15, 2017).

[40] Zhou Y Zhou B Pache L Chang M Khodabakhshi AH Tanaseichuk O. Metascape provides a biologist-oriented resource for the analysis of systems-level datasets. Nat Commun. (2019) 10:1523. 10.1038/s41467-019-09234-6 30944313 PMC6447622

[41] Walter W Sánchez-Cabo F Ricote M. GOplot: An R package for visually combining expression data with functional analysis. Bioinformatics. (2015) 31:2912–14. 10.1093/bioinformatics/btv300 25964631

[42] Foroushani ABK Brinkman FSL Lynn DJ. Pathway-GPS and SIGORA: identifying relevant pathways based on the over-representation of their gene-pair signatures. PeerJ. (2013) 1:e229. 10.7717/peerj.229 24432194 PMC3883547

[43] Xia J Benner MJ Hancock REW. NetworkAnalyst - integrative approaches for protein–protein interaction network analysis and visual exploration. Nucleic Acids Res. (2014) 42:W167–74. 10.1093/nar/gku443 24861621 PMC4086107

[44] Xia J Gill EE Hancock REW. NetworkAnalyst for statistical, visual and network-based meta-analysis of gene expression data. Nat Protoc. (2015) 10:823–44. 10.1038/nprot.2015.052 25950236

[45] Breuer K Foroushani AK Laird MR Chen C Sribnaia A Lo R. InnateDB: systems biology of innate immunity and beyond—recent updates and continuing curation. Nucleic Acids Res. (2013) 41:D1228–33. 10.1093/nar/gks1147 23180781 PMC3531080

[46] Oliver BGG Lim S Wark P Laza-Stanca V King N Black JL. Rhinovirus exposure impairs immune responses to bacterial products in human alveolar macrophages. Thorax. (2008) 63:519–25. 10.1136/thx.2007.081752 18245149

[47] Stokes CA Kaur R Edwards MR Mondhe M Robinson D Prestwich EC. Human rhinovirus-induced inflammatory responses are inhibited by phosphatidylserine containing liposomes. Mucosal Immunol. (2016) 9:1303–16. 10.1038/mi.2015.137 26906404 PMC4883656

[48] Singanayagam A Loo S-L Calderazzo M Finney LJ Trujillo Torralbo M-B Bakhsoliani E. Antiviral immunity is impaired in COPD patients with frequent exacerbations. Am J Physiol Lung Cell Mol Physiol. (2019) 317:893–903. 10.1152/ajplung.00253.2019 31513433 PMC6962603

[49] He JQ Sandford AJ Wang IM Stepaniants S Knight DA Kicic A. Selection of housekeeping genes for real-time PCR in atopic human bronchial epithelial cells. Eur Respir J. (2008) 32:755–62. 10.1183/09031936.00129107 18417509

[50] O'neal WK Knowles MR. Cystic fibrosis disease modifiers: complex genetics defines the phenotypic diversity in a monogenic disease. Annu Rev Genom Hum Genet. (2018) 19:201–22. 10.1146/annurev-genom-083117-021329 29709203

[51] Cesur MF Durmuş S. Systems biology modeling to study pathogen–host interactions. Methods Mol Biol. (2018) 1734:97–112. 10.1007/978-1-4939-7604-1_10 29288450

[52] Yogarajah T Ong KC Perera D Wong KT. AIM2 inflammasome-mediated pyroptosis in enterovirus A71-infected neuronal cells restricts viral replication. Sci Rep. (2017) 7:5845. 10.1038/s41598-017-05589-2 28724943 PMC5517550

[53] Rosebeck S Leaman DW. Mitochondrial localization and pro-apoptotic effects of the interferon-inducible protein ISG12a. Apoptosis. (2008) 13:562–72. 10.1007/s10495-008-0190-0 18330707

[54] Ho HH Ivashkiv LB. Role of STAT3 in type i interferon responses. J Biol Chem. (2006) 281:14111–18. 10.1074/jbc.M511797200 16571725

[55] Wang W-B Levy DE Lee C-K. STAT3 negatively regulates type I IFN-mediated antiviral response. J Immunol. (2011) 187:2578–85. 10.4049/jimmunol.1004128 21810606

[56] Montgomery ST Mall MA Kicic A Stick SM. Hypoxia and sterile inflammation in cystic fibrosis airways: mechanisms and potential therapies. Eur Respir J. (2017) 49:1600903. 10.1183/13993003.00903-2016 28052955

[57] Montgomery ST Dittrich AS Garratt LW Turkovic L Frey DL Stick SM. Interleukin-1 is associated with inflammation and structural lung disease in young children with cystic fibrosis. J Cyst Fibros. (2018) 17:715–22. 10.1016/j.jcf.2018.05.006 29884450

[58] Kieninger E Vareille M Kopf BS Blank F Alves MP Gisler FM. Lack of an exaggerated inflammatory response on virus infection in cystic fibrosis. Eur Respir J. (2012) 39:297–304. 10.1183/09031936.00054511 21719483

[59] Ribeiro CMP Paradiso AM Carew MA Shears SB Boucher RC. Cystic fibrosis airway epithelial Ca2+ I signaling: the mechanism for the larger agonist-mediated Ca2+ I signals in human cystic fibrosis airway epithelia. J Biol Chem. (2005) 280:10202–9. 10.1074/jbc.M410617200 15647273

[60] Tarran R Button B Picher M Paradiso AM Ribeiro CM Lazarowski ER. Normal and cystic fibrosis airway surface liquid homeostasis. J Biol Chem. (2005) 280:35751–9. 10.1074/jbc.M505832200 16087672 PMC2924153

[61] Wetmore DR Joseloff E Pilewski J Lee DP Lawton KA Mitchell MW. Metabolomic profiling reveals biochemical pathways and biomarkers associated with pathogenesis in cystic fibrosis cells. J Biol Chem. (2010) 285:30516–22. 10.1074/jbc.M110.140806 20675369 PMC2945545

[62] Tiringer K Treis A Fucik P Gona M Gruber S Renner S. A Th17- and Th2-skewed cytokine profile in cystic fibrosis lungs represents a potential risk factor for *Pseudomonas aeruginosa* infection. Am J Respir Crit Care Med. (2013) 187:621–9. 10.1164/rccm.201206-1150OC 23306544

[63] Bortolotti P Hennart B Thieffry C Jausions G Faure E Grandjean T. Tryptophan catabolism in *Pseudomonas aeruginosa* and potential for inter-kingdom relationship. BMC Microbiol. (2016) 16:137. 10.1186/s12866-016-0756-x 27392067 PMC4938989

[64] Rhim AD Kothari VA Park PJ Mulberg AE Glick MC Scanlin TF. Terminal glycosylation of cystic fibrosis airway epithelial cells. Glycoconj J. 17:385–91. 10.1023/A:1007156014384 11294504

[65] Glick MC Kothari VA Liu A Stoykova LI Scanlin TF. Activity of fucosyltransferases and altered glycosylation in cystic fibrosis airway epithelial cells. Biochimie. (2001) 83:743–7. 10.1016/S0300-9084(01)01323-2 11530206

[66] Stoykova LI Liu A Scanlin TF Glick MC. α1,3Fucosyltransferases in cystic fibrosis airway epithelial cells. Biochimie. (2003) 85:363–7. 10.1016/S0300-9084(03)00061-0 12770774

[67] Virella-Lowell I Herlihy J-D Liu B Lopez C Cruz P Muller C. Effects of CFTR, interleukin-10, and *Pseudomonas aeruginosa* on gene expression profiles in a CF bronchial epithelial cell Line. Mol Ther. (2004) 10:562–73. 10.1016/j.ymthe.2004.06.215 15336656

[68] Linden SK Sutton P Karlsson NG Korolik V McGuckin MA. Mucins in the mucosal barrier to infection. Mucosal Immunol. (2008) 1:183–97. 10.1038/mi.2008.5 19079178 PMC7100821

[69] Hsu J-L Chiang P-C Guh J-H. Tunicamycin induces resistance to camptothecin and etoposide in human hepatocellular carcinoma cells: role of cell-cycle arrest and GRP78. Naunyn Schmiedebergs Arch Pharmacol. (2009) 380:373–82. 10.1007/s00210-009-0453-5 19777212

[70] Gualdoni GA Mayer KA Kapsch A-M Kreuzberg K Puck A Kienzl P. Rhinovirus induces an anabolic reprogramming in host cell metabolism essential for viral replication. Proc Natl Acad Sci USA. (2018) 115:E7158–65. 10.1073/pnas.1800525115 29987044 PMC6065033

[71] Gray RD Duncan A Noble D Imrie M O'Reilly DSJ Innes JA. Sputum trace metals are biomarkers of inflammatory and suppurative lung disease. Chest. (2010) 137:635–41. 10.1378/chest.09-1047 19801580

[72] Hendricks MR Lashua LP Fischer DK Flitter BA Eichinger KM Durbin JE. Respiratory syncytial virus infection enhances *Pseudomonas aeruginosa* biofilm growth through dysregulation of nutritional immunity. Proc Natl Acad Sci USA. (2016) 113:1642–7. 10.1073/pnas.1516979113 26729873 PMC4760822

[73] Jakiela B Brockman-Schneider R Amineva S Lee W-M Gern JE. Basal cells of differentiated bronchial epithelium are more susceptible to rhinovirus infection. Am J Respir Cell Mol Biol. (2008) 38:517–23. 10.1165/rcmb.2007-0050OC 18063839 PMC2358970

