# Supplementary material for: Rhinovirus Infection Drives Complex Host Airway Molecular Responses in Children With Cystic Fibrosis
Source: Front Immunol. 2020 Jul 16;11:1327. doi: 10.3389/fimmu.2020.01327 (PMC7378398; doi:10.3389/fimmu.2020.01327)
Supplement: Supplementary file 1 [file Data_Sheet_1.docx]

Supplementary Material

# Supplementary Material and Methods

## Reverse-Transcription Polymerase Chain Reaction (RT-PCR)

Total RNA extracted from experimental samples were initially synthesised to cDNA using a 4 MultiSribe™ Reverse Transcription Kit (Applied Biosystems, Foster City,CA, USA). Here, 5 samples were added at 200 ng of RNA to a mixture of 1 x reverse transcriptase buffer, dNTP 6 (100 μM), MgCl2 (1.1 mM), random hexamers (10 μM), RNase inhibitor (10 units (U)), 7 MultiScribe™ reverse transcriptase (4 U) and RNase-free water. Samples were then placed in a 8 thermal cycler and run on a standard reverse transcription program of 25°C for 10 min, 48°C for 9 60 min and 95°C for 5 min.

## Real Time Quantitative PCR (qPCR)

Gene expression of target genes and housekeeping genes in CF and non-CF primary airway epithelial cells were assessed using real time qPCR. Pre-designed Taqman™ primer/probes with fluorescein amidite (FAM) labels were selected and are listed in supplementary Table S8. The reaction consisted of 5 μL of cDNA template, 5 μL of Taqman™ Universal PCR Master Mix buffer and 0.5 μL of Taqman™ primer/probes and RNase-free water. Real time qPCR reactions were carried out in 2 technical replicates. Cyclophilin A (PPIA) was used as the housekeeping gene due to its stable expression across different primary bronchial epithelial cells (He et al., 2008). The real time PCR reaction was performed using a Quanstudio™ 7 Flex consisted of 1 cycle of °C for 2 min, followed by one cycle of 95 °C for 10 min to activate polymerase, and followed by 40 alternating cycles of denaturation at 95 °C for 15 sec and of annealing and elongation at 60 °C for 1 min. Expression of target genes was determined as ΔCT values, relative to endogenous control and expression of housekeeping gene, PPIA.

# Supplementary Figures and Tables

## Supplementary Figures

**Supplementary Figure 1****.** Dot plot illustrating the total sequence of post alignment mapping quality above 30 (B); percentage of post alignment mapping score above 30. Data points represent individual samples for healthy controls (turquoise), healthy infected (purple), CF control (coral) and CF infected (green).

# A.

**
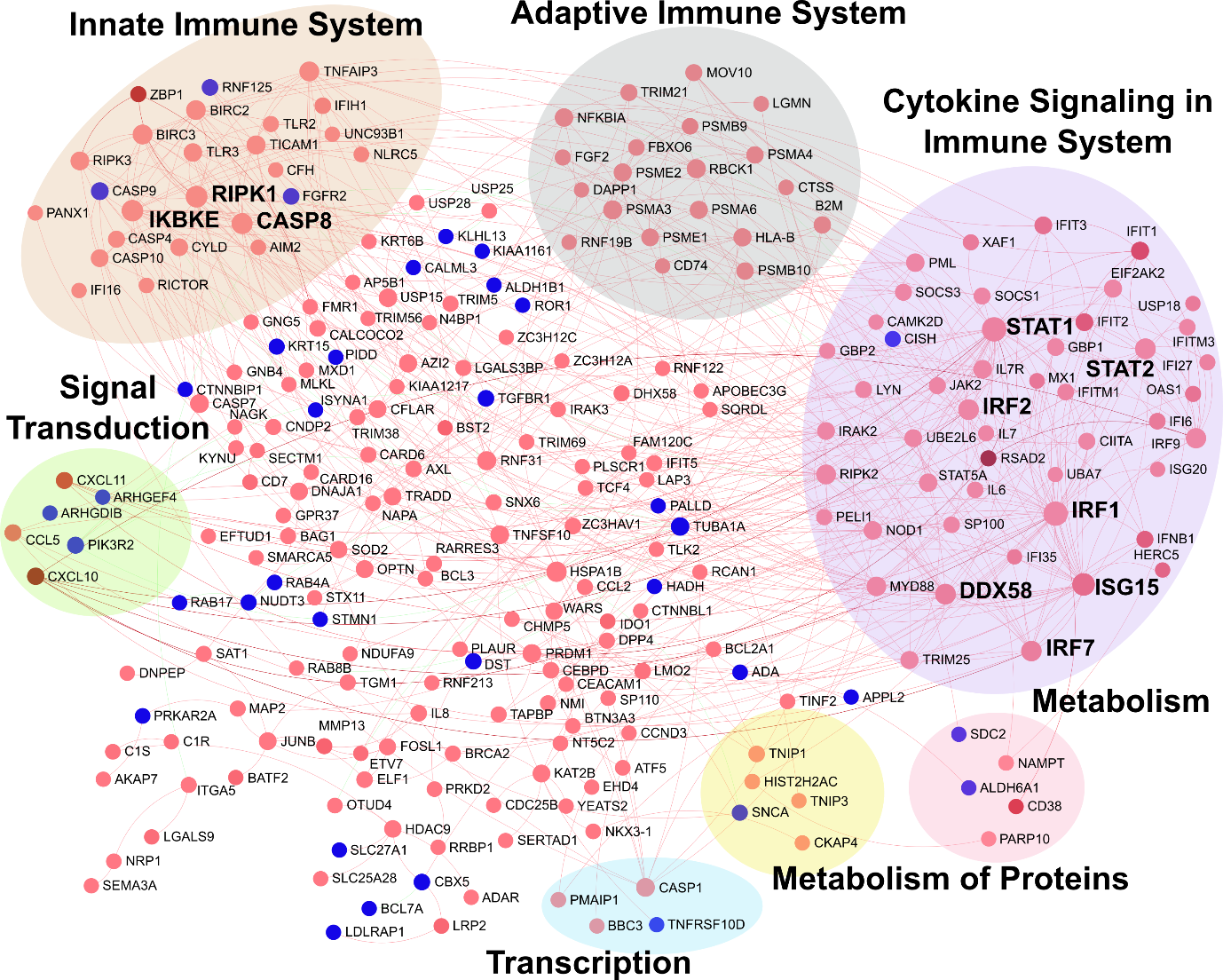
**


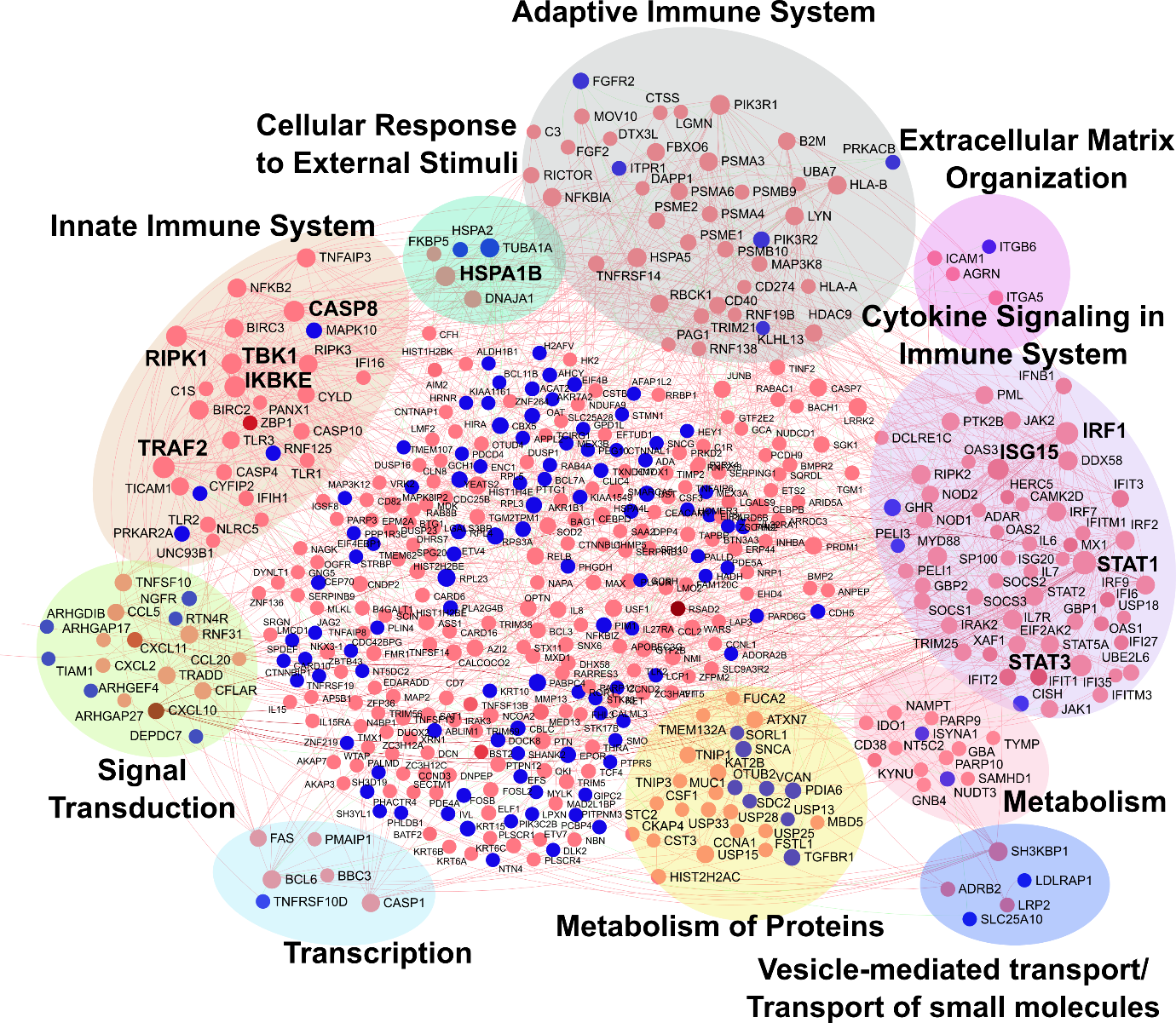
B.

**Supplementary Figure 2.** The protein-protein interaction (PPI) network for non-CF response (A) and CF response (B) were generated using NetworkAnalyst, the proteins/genes were denoted with “nodes” and the interaction between any two proteins/genes was denoted by “edge.” The network displayed is “continent” (Subnetwork 1). Red and green color nodes represent upregulated and downregulated differentially expressed genes, respectively. Both non-CF (B) and CF (C) response are grouped according to function including adaptive immune system, innate immune system, cytokine signaling in immune system, metabolism or biosynthetic pathways and signal transduction. The top 10 key hub genes for both subnetworks were colored in bold. The key hub genes for non-CF and CF response are mainly interconnected with genes involved in cytokine signaling and immune response, such as *STAT1, STAT2, IRF1, IRF2, IRF7, DDx58, ISG15, RIPK1,IKBKE* *and CASP8* for non-CF response; *STAT1, STAT3, IRF1, ISG15, RIPK1, TBK1,CASP8, IKBKE, HSPA1B* and *TRAF2*.


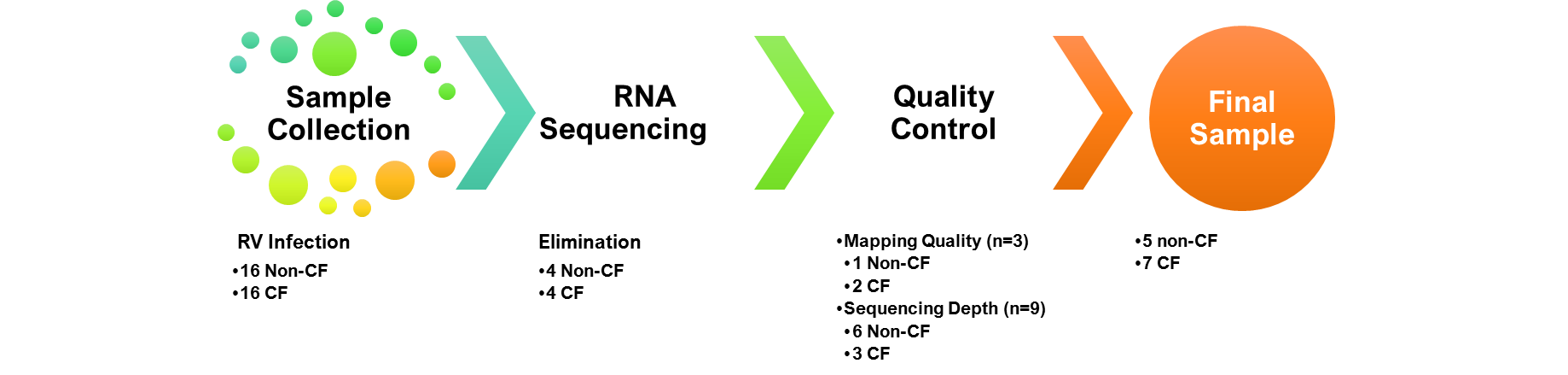


**Supplementary Figure 3.** *In vitro* RV infection experiments were performed on primary AECs derived from 32 children (16 non-CF and 16 CF). RNA-Sequencing was performed on all samples and an exclusion process implemented as part of stringent quality control. Eight samples were initially eliminated since these were run on a separate sequencer. Additional samples were then excluded from analysis for failing to have a (i) mapping quality score greater than 30, and (ii) sequencing depth of less than one million reads. Transcriptomic analysis was finally performed on 7 CF and 5 non-CF children using DESeq2 and applying a cutoff of ≥ 1.5-fold for foldchange in gene expression.
